# A more complete picture: capturing single nucleotide variant diversity in extended-spectrum beta-lactamase producing Escherichia coli using post-enrichment metagenomics

**DOI:** 10.1099/mgen.0.001757

**Published:** 2026-06-22

**Authors:** Sarah Gallichan, Tommi Mäklin, Esther Picton-Barlow, Claudia McKeown, Sally Forrest, Jukka Corander, Maria Moore, Nicholas A. Feasey, Eva Heinz, Fabrice E. Graf, Joseph M. Lewis

**Affiliations:** 1 The School of Medicine, University of St Andrews, St Andrews, UK; 2 Department of Biostatistics, University of Oslo, Oslo, Norway; 3 Department of Clinical Sciences, Liverpool School of Tropical Medicine, Liverpool, UK; 4 Wellcome Sanger Institute, Hinxton, UK; 5 Department of Genetics, University of Cambridge, Cambridge, UK; 6 Department of Vector Biology, Liverpool School of Tropical Medicine, Liverpool, UK; 7 Strathclyde Institute of Pharmacy & Biomedical Sciences, University of Strathclyde, Glasgow, UK

**Keywords:** antimicrobial resistance, bioinformatic methods, molecular epidemiology, transmission surveillance

## Abstract

Inferring transmission relies on accurately distinguishing between isolates from the same source and those from different sources, and high-quality genomic data are frequently used to model transmission scenarios. The post-enrichment metagenome sequencing (pe-MGS) method uses a sequencing approach to analyse the diversity of a target pathogen enriched by pre-culturing and has been effectively used to analyse the transmission of nosocomial infections. However, a direct comparison of single nucleotide variant (SNV) call accuracy, cost and feasibility between single-colony whole-genome sequence (sc-WGS) data and pe-MGS for an antimicrobial resistant bacteria of clinical importance, extended-spectrum beta-lactamase producing *Escherichia coli* (ESBL-EC), is required for implementation in large-scale clinical studies. A spiked stool sample and rectal swabs from six study participants were pre-enriched in buffered peptone water and cultured on MacConkey agar with 1 mg l^−1^ cefotaxime. Seven single colonies were picked, and the remaining biomass of all colonies was collected from each plate, sequenced and analysed using the mSWEEP/mGEMS pipeline. We created a custom SNV calling workflow that allows heterozygous SNVs in a bacterial population and found that the choice of reference changed the number of measurable SNV distances between the sc-WGS and pe-MGS. Using our custom workflow with a core-gene reference captured 99% of all the SNV calls from multiple sc-WGS data in the pe-MGS data of the same culture. The plate sweep method offers a feasible, cost-effective alternative to multiple single colony picks for describing within-host ESBL-EC diversity. The workflow we developed allows for effective SNV calling from pe-MGS data that were comparable to SNV calls from multiple sc-WGS data from the same sample.

**Impact Statement**

For bacterial species with high within-patient diversity and within-genome variation, such as the opportunistic pathogen *Escherichia coli*, capturing the full diversity is essential to identify transmission events. Pre-enriching the species of interest from patient samples, and then sequencing all colonies, post-enrichment metagenomics (pe-MGS) promises to be a cost-effective, efficient method for capturing the full diversity. To estimate transmission events with high confidence and make it applicable for hospital transmission studies, single nucleotide variants (SNVs) have to be identified with an equivalent resolution as would be achieved when using single-colony whole-genome sequencing (sc-WGS) on all colonies. Here, we present a proof-of-concept study on a set of stool samples and rectal swabs from healthy participants, where we developed a new workflow tested against these control samples. All samples were analysed using both sc-WGS and pe-MGS from the same plate, following pre-enrichment for the species and phenotype of interest (drug-resistant *E. coli*). This direct comparison allowed us to assess the reconstruction of SNVs between the two approaches on clinically relevant sample types at the highest resolution. We show that by using a newly developed SNV calling workflow, a core-gene reference allowed us to identify comparable SNVs in the pe-MGS to the sc-WGS sequence data. The pe-MGS offers a cheaper and time-saving alternative to multiple sc-WGS and thus has the potential to be integrated into public health settings for routine surveillance in the future, such a roll-out will be dependent on further validation studies with more diverse samples and/or strains and matrices.

## Data Summary

The short-read sequence data generated in this study have been submitted to the European Nucleotide Archive (ENA, https://www.ebi.ac.uk/ena/browser/view/PRJEB101999), and their individual accession numbers are listed in Table S1. Github repository: https://github.com/joelewis101/TRACS-liverpool/tree/main/bioinformatics . All protocols developed and utilized in this study have been detailed and provided in the article and supplementary data files.

## Introduction

Identifying bacterial pathogen transmission events is important to understand spread, highlight those at risk of infection and implement targeted measures to prevent further infection [1]. In order to infer pathogen transmission, it is necessary to identify the presence or absence of differences between isolates, a process traditionally called ‘typing’. Typing methods rely on accurately and unambiguously distinguishing between isolates from the same source and those from different sources [2]. Genomic data provide a high-resolution method to type pathogens by measuring the genetic distance of pathogens at single nucleotide variant (SNV) resolution. This requires sequence data with sufficient read quality and depth to discern between highly similar strains [3,4]. Given the still substantial cost of sample preparation and WGS at the necessary read depth and quality, transmission studies often rely on sequencing a single bacterial colony from each sample which gives high resolution but no information of the diversity [5,7].

For pathogens that exhibit largely clonal outbreaks and are not part of the healthy human microbiome, such as *Salmonella enterica* serovar Typhi*,* single-colony whole-genome sequencing (sc-WGS) is often sufficient to capture transmission events [8]. However, for highly diverse opportunistic pathogens that are also part of a healthy microbiome, such as *Escherichia coli*, the chance of capturing transmission events with a single colony from complex microbial samples becomes more unlikely as there is substantial within-sample diversity, within which the strain of interest has to be identified. Studies of human stool samples suggest that while the analysis of five *E. coli* colonies from a sample can detect the dominant genotype at >99% probability, as many as 28 single colonies per stool sample are required to detect minor genotypes (at 90% probability) [9,10]. However, sequencing and analysis of multiple single colony picks to cover the diversity and detect low-abundance strains is time-consuming, expensive and not practical in routine transmission studies. For *E. coli*, a bacterium that is spread by the faecal-oral route where gut colonization generally precedes infection, and which is used as a marker of bacterial transmission [11] (i.e. by water, sanitation and hygiene specialists), pragmatic approaches to describing full *E. coli* diversity within stool samples, at scale, are needed.

Metagenomic approaches offer a potential alternative to capture the full microbial diversity within a complex sample like stool [12]. While shotgun-metagenomics on direct DNA extractions from clinical samples has been used in clinical settings to identify pathogens, it may have limited success, or require expensive ultra-deep sequencing, to detect bacteria present at low abundances in complex microbial communities. This is often the case with *E. coli* in human stool samples, which generally are in abundance of the order of 1% within the human gut microbiome [13,14]. Thus, addressing the within-species diversity of *E. coli* in stool using shotgun-metagenomic sequencing is currently not feasible without sequencing depths that are prohibitively expensive. To strike a balance between the limited single colony analysis and the full microbial diversity of a shotgun-metagenomic approach, a variety of novel approaches, variously termed plate-sweep metagenomics, limited diversity metagenomics and post-enrichment metagenomics (pe-MGS) have been proposed to resolve these issues. For the purposes of this manuscript, we will use the term pe-MGS.

These approaches typically start with a selective enrichment step to enrich for the bacteria of interest (e.g. *E. coli*) in broth, or an agar plate or both, overcoming the challenge of the low abundance of the organism of interest in raw stool. It proceeds by using all grown colonies (e.g. plate sweep, the picking of all colonies from a selective culture plate) for one combined DNA extraction and a single metagenomics sequencing run, and subsequent *in silico* reconstruction of the unique strains present in the sample [15]. The advantage of this strategy is the removal of DNA from host or bacteria not of interest, which are in a high proportion in stool samples, allowing for specific focus on the species of interest or drug-resistant phenotype, and the potential for a full description of bacterial diversity at much reduced labour and sequencing cost. The plate sweep method has been effectively used to analyse the within-sample diversity of several pathogens [16,17], including the transmission of community-acquired *Streptococcus pneumoniae,* as well as nosocomial infections *Klebsiella pneumoniae, E. coli, Enterococcus* species*, Pseudomonas aeruginosa* and *Staphylococcus aureus*, in a clinical setting [18,20].

In this proof-of-concept study, we assessed the feasibility of describing the diversity of extended-spectrum beta-lactamase producing *E. coli* (ESBL-EC) in stool samples using pe-MGS and the mSWEEP/mGEMS [15] workflow. ESBL-EC are resistant to aminopenicillins and third-generation cephalosporin antibiotics, which are commonly used as the first-line treatment for Gram-negative bacterial infections. Infections with ESBL-EC result in higher morbidity and mortality and longer hospital stays [21,23]. Since most transmission and surveillance studies rely on sequencing of single colonies, the within-host diversity of ESBL-EC has not been established. Here, we compare the pe-MGS to the gold standard of sc-WGS to detect differences between highly related ESBL-EC strains in order to inform a healthcare-associated ESBL-EC transmission study. We compare the predicted ESBL-EC diversity from human stool and rectal swabs based on the SNV call accuracy, cost and feasibility between these approaches.

## Methods

### Sample preparation

Stool from three healthy adult volunteers (individuals not on antibiotics or experiencing any gastrointestinal problems) was mixed and a slurry created for use as a model stool sample (LSTM Research Tissue Bank RTB/2022/007). A spiked stool sample was created with two clinical ESBL-EC strains (a ST167 and ST131, previously isolated from human stool samples in Malawi) [17] mixed at an equal ratio at 10^4^ c.f.u. ml^−1^, 10 µl spiked into 1 ml of pre-heated stool slurry (37 °C) and vortexed thoroughly. Rectal swabs were collected from adult study participants (≥18 years) residing in hospitals and care homes in Liverpool as part of the TRACS-Liverpool study (TRACS: 22/NW/0343) [24]. The spiked stool and rectal swabs were then cultured according to an optimized method to recover ESBL-EC [25]. Briefly, stool and rectal swabs were pre-enriched for 4 h in buffered peptone water (BPW) at 37 °C while shaking at 220 r.p.m., 10 µl of enriched culture was then spread on cefotaxime (1 mg l^−1^) supplemented MacConkey agar and incubated at 37 °C overnight. To our knowledge, the optimal number of colony picks to detect all the colonizing ESBL-EC genotypes within a human gut has not been determined. To increase the probability of detecting multiple ESBL-EC genotypes, seven single colonies were picked using a 1 µl inoculation loop, spread on separate cefotaxime-supplemented MacConkey agar plates (single colony picks) and incubated at 37 °C overnight. The remaining biomass was then scraped off using a 10 µl inoculation loop and transferred the collected culture into a 1.5 ml tube (plate sweep) containing PBS (multiple scrapes were required for cultures with a large amount of biomass) and extracted using the MasterPure Complete DNA and RNA Purification Kit (Lucigen, Wisconsin, USA) according to the manufacturer’s recommendations. The same was done for the extraction of the single-colony-derived plates the following day.

### Sequencing

The DNA concentration and integrity were measured using the Qubit [26] and the TapeStation System [27], respectively. The extracted DNA from the single colony plates and the plate sweeps was then sent for Illumina sequencing, at depths of 1 Gb and 6 Gb, respectively, to Azenta Life Sciences (Frankfurt, Germany). Data were obtained as paired-end 150 base pair reads. Adaptor sequences were removed using fastp version 0.23.4 [28], and the quality of the trimmed reads was assessed using the quality metrics provided by FastQC version 0.11.9 [29].

### Bioinformatic analysis

Reconstruction of bacterial strains within the sc-WGS and pe-MGS was done using the mSWEEP (v 2.2.0) and mGEMS (v 1.3.3) algorithms [15,16], using a curated reference database from a previous study [30,31] (available from Zenodo) [32] consisting of 14,438 *E. coli* assemblies. The sample reads were first pseudoaligned to the reference database using Themisto (v 3.2.2) [33], and then mSWEEP was used to calculate the likelihood and relative abundance estimates of each reference PopPUNK (version 2.6.3) [31] cluster being in the sample using the pseudoalignment results. The sample reads were then binned by running the mGEMS algorithm from mSWEEP with the –bin-reads option. The mGEMS read bins for each strain were quality controlled using the demix_check tool (v 1.0) [34] and scored (1 – highest confidence, 4 – lowest confidence) based on the mash (v 2.0) [35] distance between the binned reads and the sequences in the reference database. These samples were then extracted from the relevant read bins using mGEMS for use in SNV analysis. Multi-locus sequence typing was done using ARIBA (v2.11.1) [36,37] with the seven-gene Achtman scheme [38].

### Reference genomes

A closely related, Phylogroup B2, ST131 *E. coli* complete genome (Accession number: NC_000913, plasmids removed); a distantly related, Phylogroup F, ST1485 *E. coli* complete genome (Accession number: NZ_CP042896, plasmids removed); and an *E. coli* core-gene list were used as references. To create an *E. coli* core-gene list for use as a reference, the genes present in 99% of a pan-genome from a previously curated collection of 10,000 *E. coli* genomes [39] were extracted from the pan-genome using SeqKit version 2.8.2 [40], resulting in two 384 genes, with a total length of 2.29 Mb.

### SNV calling workflow

The sc-WGS were mapped to references, and the SNVs were called using snippy version 4.3.6 (https://github.com/tseemann/snippy). A core genome alignment of all the single-pick sequences was created using snippy-core (with default settings). All non-nucleotide characters, such as ‘n’, were removed from the multisequence alignment using the snippy-clean_full_aln, and the resulting SNVs were extracted using snp-sites (with the -c option to output exclusively columns containing ATCG). A pairwise SNV distance comparison between each of the single picks in the sample was then created using snp-dists version 0.8.2 (https://github.com/tseemann/snp-dists). To account for the possibility of heterozygous SNVs present in mixed samples in the sweep samples, we modified the settings of snippy for these samples as follows: the SNVs were called using freebayes version 1.3.6 [41] (using the --pooled-continuous option) from the binary alignment map (BAM) file as produced by snippy using bwa-mem. The raw variant call files were filtered to include all variant types with a phred-scored quality of more than 100 (QUAL >100), a depth of at least 20 (DP>=20), and supported by at least 85% of reads (A0/DP >0.85) at a given site using bcftools version 1.20 [42]. In addition, a sliding window approach was used to mask regions that had clusters of SNV calls, which could be sites of recombination or bacteriophage inserts that would influence the accuracy of transmission inference. The sliding window worked according to an algorithm [37] whereby the rate of SNVs within an alignment was calculated within a set window (window size=length of reference genome/total number of SNVs) and if a significant number of SNVs (*P*=0.05) were captured in the window, then those SNVs were excluded. Sites with low quality SNV calls (QUAL <100) and/or had low sequencing depth (DP <20) were masked across all SNV calls when comparing SNP distances. Heterozygous variant types (GT not equal to 1/1) were masked in sc-WGS. This SNV calling process was condensed into a Snakemake (version 8.29.2) [43] workflow that is accessible from the TRACS-Liverpool Github page (https://github.com/joelewis101/TRACS-liverpool/tree/main/bioinformatics/snakemake).

### Sequence depth

To identify the optimum, we simulated different sequencing depths by subsampling reads; the number of reads that approximated 10×, 15×, 20×, 25×, 30×, 100× and 500× sequence depth (Table S2) was randomly subsampled using Seqtk (version 1.4.) [44] from the plate sweep sequence data. The subsampled sequence data were then analysed through the mSWEEP/mGEMS pipeline and the custom SNV calling workflow using the core *E. coli* gene list as a reference. To estimate the plate sweep sequence depth that can be used to accurately capture 99% of the combined single pick SNV calls, a logistic regression model was fitted to the rarified plate sweep sequence data using the nlstools R package version 2.1–0 [45].

## Results

### Calibrating a workflow to analyse SNV diversity in plate sweeps

**Fig. 1.**
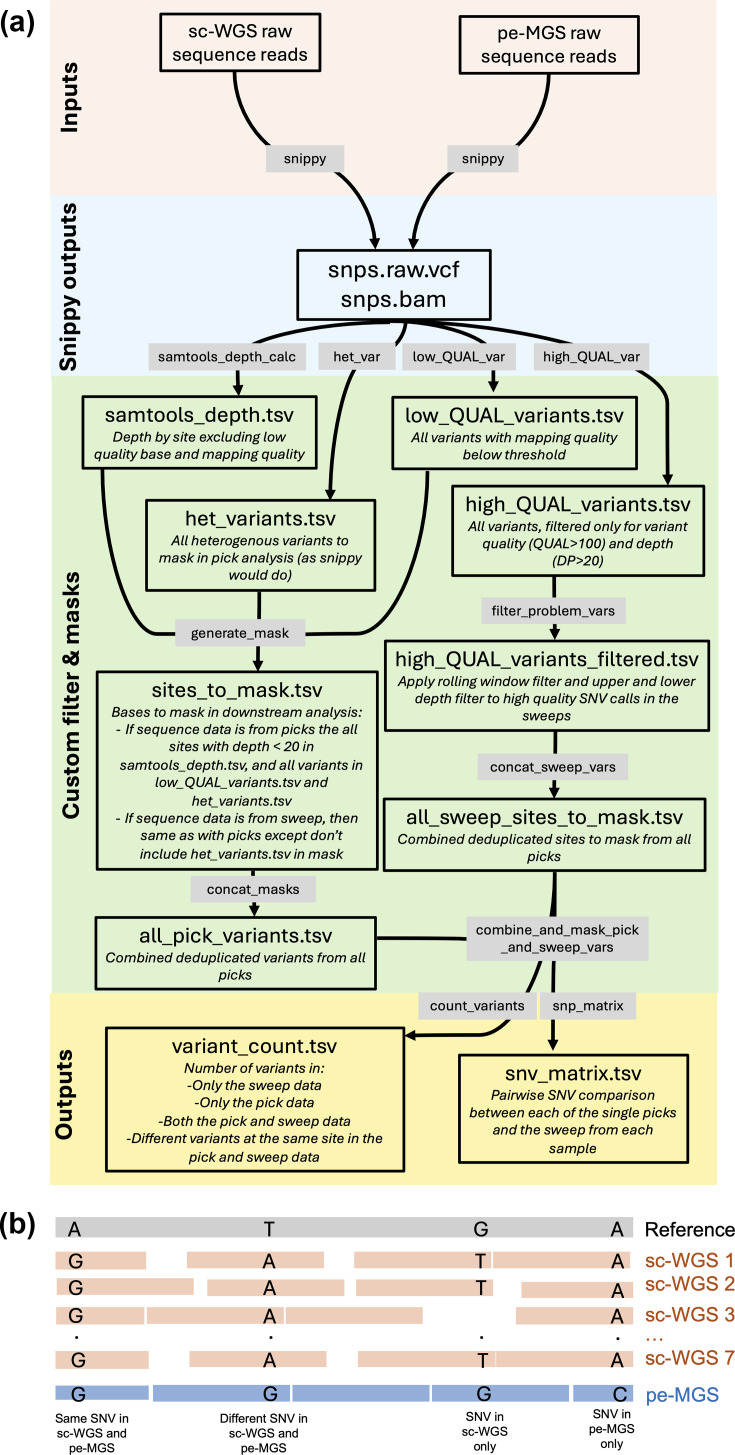
(**a**) Overview of the custom SNV calling workflow that directly compares the SNV calls in the sequencedata of multiple sc-WGS and a pe-MGS from the same sample. Grey boxes correspond with rules in the Snakemake workflow. (**b**) Graphic illustration of the comparison of sequence reads (colour blocks) from multiple sc-WGS and a pe-MGS from the same sample. The black letters indicate SNVs in each of the sequence types, and the descriptions below indicate how the SNV comparisons were grouped in the final variant count output from the workflow.

Since tools to identify SNVs in bacteria are designed for homozygous samples and not to identify variants in mixed samples, heterozygous SNVs, as present in the plate sweep sequence data, were considered errors by default by tool, expecting input from a homozygous population as standard for bacteria WGS. Therefore, we developed a custom SNV calling workflow that allows calling the heterozygous SNVs in sweep sequence data and directly compares the SNV calls in the sweep data to the pooled single pick data (Fig. 1).

**Fig. 2.**
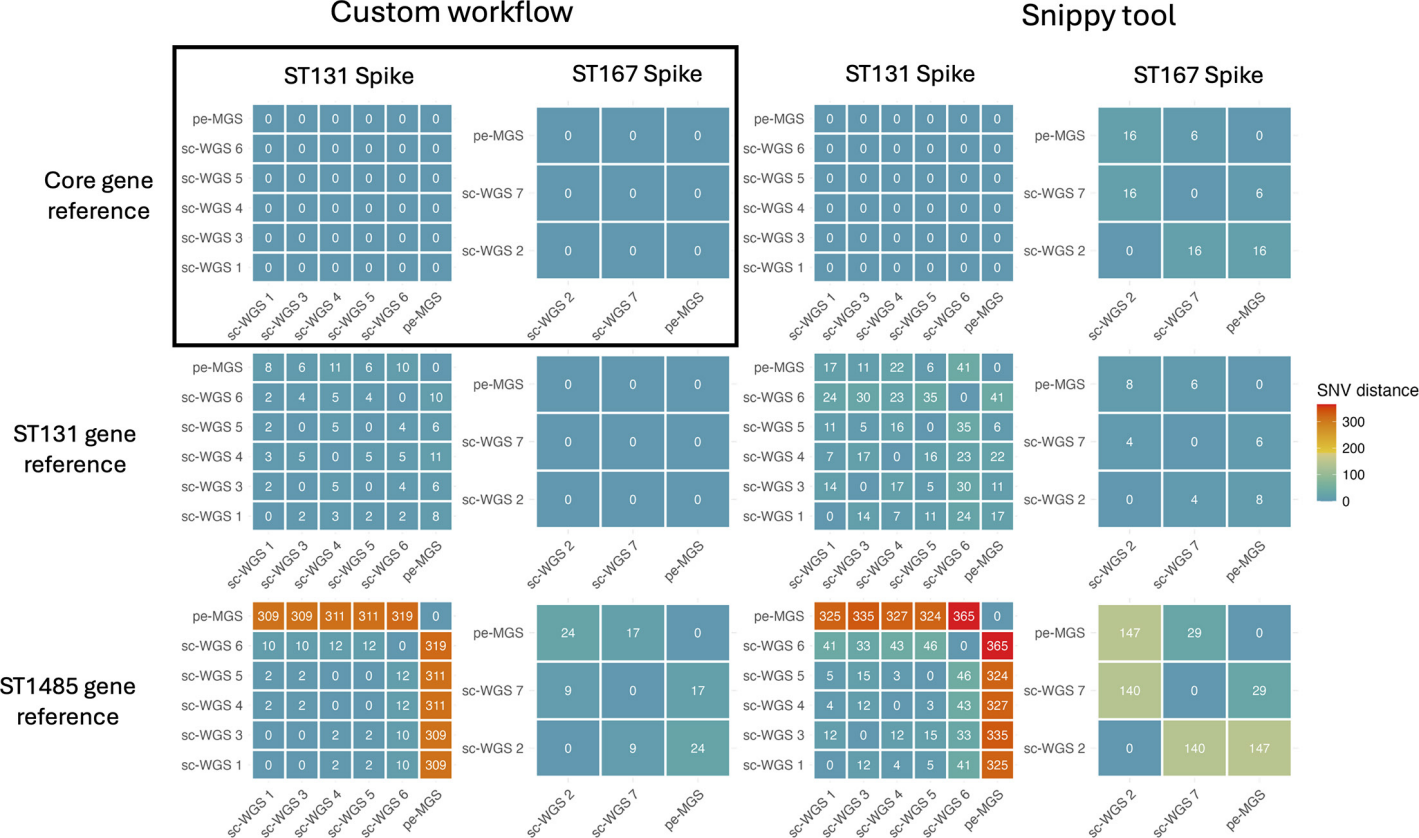
SNV distance matrices for the two ESBL-EC STs (five single colonies were ST131 and two single colonies were ST167 with corresponding sweep reads for each strain) in the spiked stool sample comparing snippy and our custom SNV calling workflow with three different references (Core-genes, ST131 and ST1485). The grey block highlights the workflow and reference that resulted in no measurable SNV distances as expected.

To calibrate this custom workflow, we used sc-WGS and sweep sequence data from a spiked stool sample. We added a mix of two well-characterized clinical ESBL-EC strains (ST167 and ST131) that we assumed were clonal and would result in no measurable SNV distances between sc-WGS and pe-MGS data for each strain. We then directly compared the SNV calls using the Snippy tool and our custom SNV calling workflow in the sc-WGS and corresponding pe-MGS data from each of the two ESBL-EC strain spikes using three different references (a core-gene reference and two complete genome references, *E. coli* ST131 and ST1485) (Fig. 2). Overall, the SNV calls differed according to the reference used, with the highest number of SNVs calls when the ST1485 reference was used in both the custom SNV calling workflow and the Snippy tool (Fig. 2). Only the custom SNV calling workflow with the core gene reference produced SNV matrices with no measurable SNV distances between sc-WGS and pe-MGS from the spiked stool, as we expected (Fig. 2).

Since we assumed that the two ESBL-EC strains spiked into the stool were each clonal, we investigated why our custom workflow with the whole-genome references (ST131 and ST1485 references) resulted in variation in the sc-WGS and pe-MGS data. The SNV calls were high quality, with sufficient read depth, and not masked by the sliding window filter (Table S3). Thus, we assessed whether the variance was due to complex genomic regions, such as hypervariable or repeat regions (Table S3). The SNV calls mapped to the ST131 reference (*n*=17) were randomly spread across the genome and the majority of the calls, except one variant that mapped to an inner membrane protein region, did not have any associated protein data. However, the SNV calls mapped to the ST1485 reference (*n*=364) were distinctly clustered in hypervariable regions, such as genes encoding adhesin and membrane proteins [46], with 319 of the SNVs clustered to only 27 genes (Table S3). To eliminate the variance due to hypervariability and the impact of different references based on the input sample going forward, we used the core gene reference for the analysis of clinical samples.

### Plate sweeps can be used to accurately capture SNV diversity in clinical samples

Rectal swabs from six TRACS-Liverpool study participants were pre-enriched and selected for ESBL-EC on cefotaxime-supplemented MacConkey agar. The resulting single colonies were submitted for sc-WGS, and remaining biomass for pe-MGS. Seven colonies were picked for each study participant where possible for sc-WGS; two participants (1 and 3) only had six and four colonies on the selective agar, respectively, which we thus selected. Sc-WGS indicated that four of the six study participants (4/6, 67%; participants 1, 2, 5 and 6) had a single ESBL-EC ST, and the remaining two study participants (2/6, 33%; participants 3 and 4) had two ESBL-EC STs (Table 1).

**Table 1.** Characteristics of the ESBL-EC recovered from each study participant

Participant	No. of colony picks	Species	Sequence type
1	6	*E. coli*	131
2	7	*E. coli*	131
3	3	*E. coli*	38
	1	*E. coli*	1193
4	2	*E. coli*	131
	5	*E. coli*	1193
5	7	*E. coli*	349
6	7	*E. coli*	38

We then compared the SNV calls from multiple sc-WGS and the pe-MGS for each ESBL-EC STs. While the SNV consensus sequences for the sc-WGS and pe-MGS produced by the snippy tool had many SNV calls that were unique either to the sc-WGS or the pe-MGS with little agreement between them (Table S4), the SNV calls for the sc-WGS and the pe-MGS resulting from our custom workflow corresponded well (Tables 2 and S5) with 856,697 SNVs identical by both methods, and 17 SNVs present in only one or different nucleotides. Five of the eight ESBL-EC STs (5/8, 62.5%) had identical SNV calls in the sc-WGS and pe-MGS. Three of the STs (3/10, 30%) had SNV calls unique to the sc-WGS data and which were not called in the pe-MGS data. One sample contained a ST (1/10, 10%) with SNVs unique to the pe-MGS data and that was not present in the sc-WGS data; and one ST had a SNV call that was present in both the sc-WGS and the pe-MGS data but was a different variant (1/10, 10%) (Table 2).

**Table 2.** Comparison of the number of SNV calls in the sc-WGS and pe-MGS data from each study participant using the custom SNV calling workflow with the core-gene reference

	Variant allele count			
**Participant**	**Pick only**	**Sweep only**	**Different SNV in pick and sweep**	**Same SNV in pick and sweep**
**Participant 1 (ST131)**	0	0	0	51,035
**Participant 2 (ST131)**	0	0	0	49,513
**Participant 3 (ST38)**	0	0	0	46,033
**Participant 3 (ST1193)**	0	0	0	52,031
**Participant 4 (ST131)**	6	6	0	53,283
**Participant 4 (ST1193)**	0	0	0	511,944
**Participant 5 (ST349)**	1	0	0	46,699
**Participant 6 (ST38)**	3	0	1	46,159

### Required sequence depth for plate sweep SNV calls

**Fig. 3.**
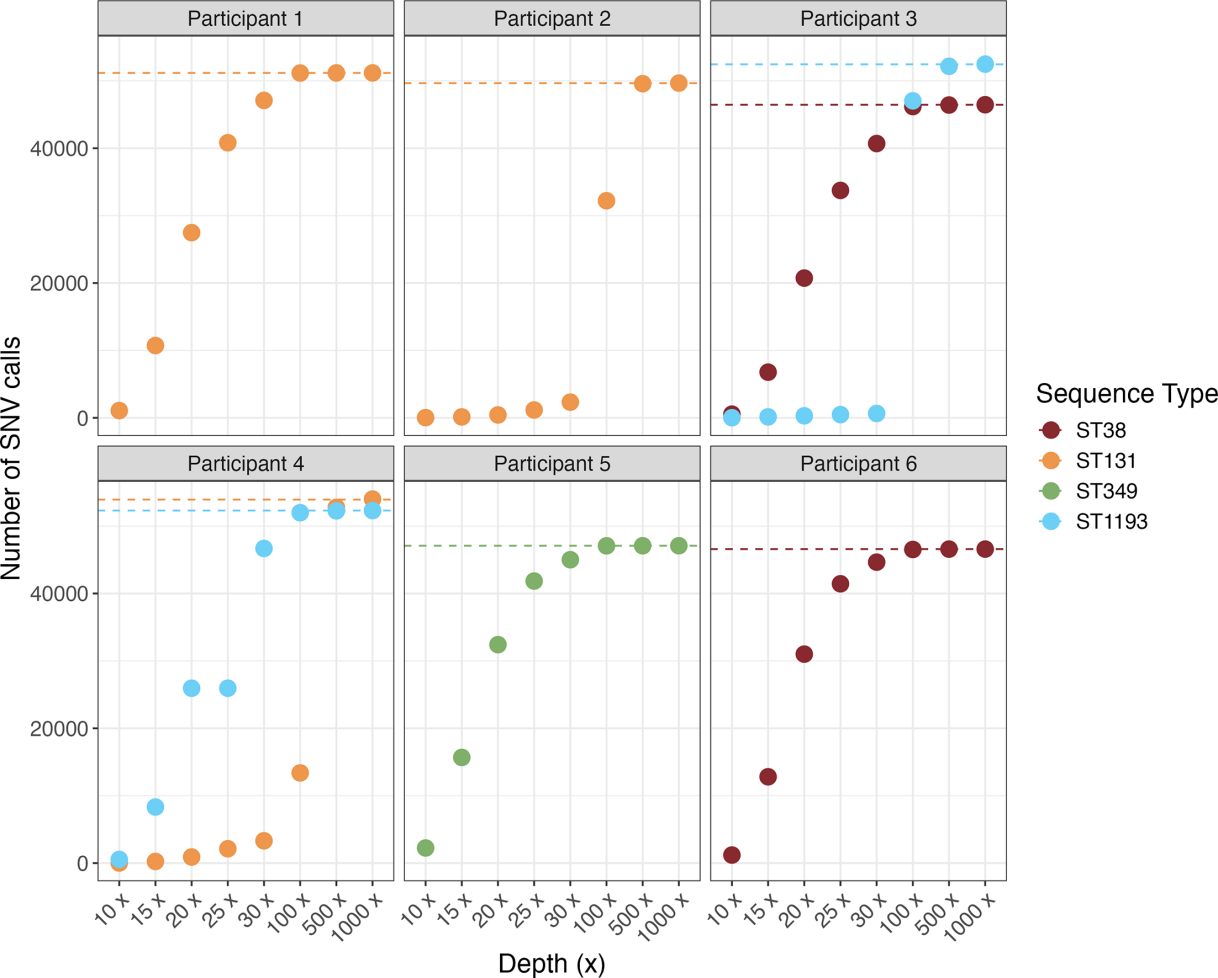
Number of SNV calls when compared to the core-gene *E. coli* reference at different sequence depths using subsampled sequence data from the plate sweeps for each participant. The horizontal dashed line represents the fixed combined single pick SNV calls for each lineage (ST) within each sample.

We sequenced the plate sweep samples at a sequencing depth of 6 Gb yielding an average of more than 1,000× coverage for the ESBL-EC strains from each participant. To approximate the sequencing depth which will allow us to capture SNV from plates sweep with a similar accuracy to SNV calls from multiple sc-WGS, we subsampled the sequence data and fitted a logistic regression model to the resulting number of sweep SNV calls (Table S6). We were able to capture 99% of all the SNV calls with 50× sequence coverage in five ESBL-EC isolates (5/8, 62.5%), 170× coverage in two ESBL-EC isolates (2/8, 25%) and 250× coverage in one ESBL-EC isolate (1/8, 12.5%) (Fig. 3).

### Plate sweep method offers a cheaper, efficient alternative to multiple colony picks for describing ESBL-EC diversity

To compare the cost of each method (sc-WGS, multiple sc-WGS and pe-MGS) for the analysis of the within-host diversity of ESBL-EC, we took into consideration the cost of the microbiology method, which includes pre-enrichment in BPW, plating on selective MacConkey agar and DNA extraction and the sequencing costs, including library preparation. The single colony pick method was the cheapest, costing around half as much as the plate sweep method (Table S7). However, when comparing the cost of multiple sc-WGS to pe-MGS, the multiple colony picks method was substantially more expensive than the plate sweep method (Table S7). Additionally, the plate sweep method required one less standard working day of microbiological processing time than single and multiple colony picks (Table S7).

## Discussion

To accurately trace the transmission of ESBL-EC*,* SNV-level analysis provides the highest resolution possible to date. Since analysis of a single colony is insufficient for capturing the full within-host ESBL-EC diversity, and metagenomics cannot provide sufficient resolution for *E. coli* within complex samples without expensive deep sequencing, the plate sweep method offers a more feasible alternative. Here, we validated the accuracy of plate sweep ESBL-EC SNV calls using a custom SNV calling workflow by directly comparing it to the SNV calls from seven single colony picks for six study participants. We demonstrated that by using our custom workflow with a core-gene reference, high-confidence SNV calls from plate sweep sequence data were equivalent to SNV calls in multiple single colony picks, and the approach reduced cost and time.

While pe-MGS has been used previously to describe SNV diversity in clinical samples [19], we present a direct comparison to SNV calls from multiple sc-WGS from the same plate. Previous analyses of within-ST diversity of clinical samples have relied on data from multiple sc-WGS to make accurate SNV calls [10,47,49] and have concluded that sequence data analysis should be able to accurately identify a difference of 10 to 17 SNVs between isolates across an *E. coli* genome [49,50]. We demonstrate here that the pe-MGS offers a cheaper, time-efficient alternative for analysing within-ST ESBL-EC diversity.

However, the accuracy of the pe-MGS SNV calls is dependent on the reference used. Complete genome references contain hypervariable regions, such as prophage regions [51], surface polysaccharides [52] and genomic islands [53] that will inevitably have an increased number of associated SNVs. Accurately captured SNV variability at these sites between two isolates does not necessarily indicate two bacteria are part of distinct transmission chains. Furthermore, these regions are challenging given their often repetitive nature, providing issues to both reference assemblies and subsequent mapping, and false-positive SNV calls have been observed in regions surrounding indels within the reference sequence, as well as in multi-copy genes that are difficult to identify and remove with quality and depth filters [54,55]. Indeed, the use of complete genome references in our study resulted in SNV calls in our double spike sample that we expected to be clonal, i.e. no SNV calls. A possible solution would be to exclude regions of hypervariability using tools, such as Dustmasker [56]. However, for our use case in the analysis of epidemiologically informative SNVs, we chose to remove any uncertainty surrounding hypervariable regions and continued with a core-gene reference that also allows for much higher reproducibility for samples with different compositions. Based on a large-scale analysis of diverse *E. coli* genomes, as it provides a reference much more equally distant to all STs regarding gene composition than a specific reference genome from a certain ST.

Additionally, we included a sliding window filter in our workflow to remove aggregated SNVs. Spatial clustering of SNVs can arise from homologous recombination, gene or genetic region (e.g. proteins with repetitive domain profiles) duplication or mobile element insertion. These SNV clusters can complicate analyses and are prone to assembly errors, as repetitive sequences are challenging to resolve particularly with short reads and subsequently are also prone to misinterpretation of transmission; thus, we chose to remove them using a sliding window approach [18].

An accurate analysis of the within-host diversity using pe-MGS has been used successfully for identification of transmission routes [18,30]. While heterozygous SNVs are not yet commonly considered when analysing bacterial phylogenetic relationships [19,30], inclusion of heterozygous SNVs has proven invaluable in transmission inference [18]. Indeed, the incorporation of SNV data into algorithms has provided accurate insights into the transmission rates in large cohort datasets [37]. Here, we observed that with the inclusion of heterozygous SNVs in the pe-MGS data analysis, the resulting within-host SNV calls closely resembled the collective SNV calls from multiple sc-WGS, reiterating the power of pe-MGS data.

While we have fully validated a workflow for SNV calling using pe-MGS data from a plate sweep, this study was limited to six rectal swab samples with a maximum of two ESBL-EC STs in two of the samples. Therefore, we do not know the SNV call accuracy, or the coverage required to accurately call SNVs in samples with three or more STs, which will need to be validated in the future. Additionally, the suggested depth for accurate SNV calling of up to two STs is highly variable and was a result of subsampling 6 Gb of sequencing data, which may not accurately reflect real sequencing biases. The use of a core-gene list as a reference is also limiting since it is not a fully assembled genome, leading to breaks in mapping at gene boundaries and requires a large collection of genomes to construct. More broadly, the pe-MGS method we assessed uses short-read sequencing and would not be expected to be able to recover mobile genetic elements like plasmids well, nor associate them with bacterial strains; thus, it is currently most useful for epidemiological purposes.

## Conclusion

Consideration of within-host diversity of ESBL-EC present in a given sample allows for a more complete picture of colonization at a certain time point. If the full diversity of ESBL-EC within the stool sample is not considered, some isolates may be misclassified as being acquired when they were present throughout the sampling period at low levels [49]. The custom SNV calling workflow we have developed here can provide a cost-effective method for defining within-host diversity at scale which will allow more accurate description of transmission events. This, in turn, will allow us to better understand transmission patterns of particular pathogens and to design, test and optimize infection prevention and control strategies to block transmission.

## Supplementary material

10.1099/mgen.0.001757 Supplementary Material 1.

## Abbreviations

BAM: binary alignment map
BPW: buffered peptone water
ENA: European Nucleotide Archive
ESBL-EC: extended-spectrum beta-lactamase producing *Escherichia coli*
pe-MGS: post-enrichment metagenome sequencing
sc-WGS: single-colony whole-genome sequencing
SNV: single nucleotide variant
